# Apoptotic proteins in *Leishmania donovani*: *in silico* screening, modeling, and validation by knock-out and gene expression analysis

**DOI:** 10.1051/parasite/2024081

**Published:** 2025-02-12

**Authors:** Ketan Kumar, Lucien Crobu, Rokhaya Thiam, Chandi C. Mandal, Yvon Sterkers, Vijay Kumar Prajapati

**Affiliations:** 1 Department of Biochemistry, School of Life Sciences, Central University of Rajasthan Ajmer 305817 India; 2 University of Montpellier, CNRS, IRD, University Hospital Center (CHU) of Montpellier, MiVEGEC, Department of Parasitology-Mycology 34295 Montpellier cedex 5 France; 3 Department of Biochemistry, University of Delhi South Campus New Delhi 110021 India

**Keywords:** Apoptosis, CRISPR-Cas9, Leishmaniasis, Molecular dynamics simulation, qRT-PCR

## Abstract

Visceral leishmaniasis, a life-threatening vector-borne illness that disproportionately affects children and elderly immunocompromised people, is a primary tropical neglected disease. No apoptotic partner proteins have yet been reported in *Leishmania donovani*, while their identification could contribute to knowledge on parasite cell death and the establishment of alternative therapeutics. We searched for mammalian Bcl-2 family protein orthologs and found one anti-apoptotic and two pro-apoptotic orthologs in *L. donovani*. A pro-death aquaporin protein, due to its characteristic BH3 domain known to interact with pro-apoptotic proteins in mammalian Bcl-2 family proteins, was also included in this study. Molecular docking and molecular dynamics simulations were conducted to assess protein-protein interactions between the identified apoptotic proteins and mimic mammalian intrinsic apoptotic pathways. The results showed that both pro-apoptotic proteins interacted with the hydrophobic pocket of the anti-apoptotic ortholog, forming a stable complex. This interaction may represent a critical event in an apoptotic pathway in *L. donovani*. To further characterise it, we used CRISPR-Cas9 approaches to target the identified proteins. Pure knocked population mutants, and episomal over-expressing mutant cells were exposed to apoptotic stimuli. Terminal deoxynucleotidyl transferase dUTP nick end labeling (TUNEL) assay and quantitative expression profiling suggested that these proteins are involved in the parasite’s apoptosis and could play a role in its survival.

## Abstract (fr)

La leishmaniose viscérale, une maladie à transmission vectorielle potentiellement mortelle qui touche de manière disproportionnée les enfants et les personnes âgées immunodéprimées, est une maladie tropicale primaire négligée. Aucune protéine partenaire apoptotique n'a encore été signalée chez *Leishmania donovani*, alors que leur identification pourrait contribuer à la connaissance de la mort cellulaire du parasite et à l'établissement de thérapies alternatives. Nous avons recherché les orthologues des protéines de la famille Bcl-2 des mammifères et avons trouvé une anti-apoptotique et deux pro-apoptotiques chez *L. donovani*. Une protéine aquaporine pro-mort, en raison de son domaine BH3 caractéristique connu pour interagir avec les protéines pro-apoptotiques des protéines de la famille Bcl-2 des mammifères, a également été incluse dans cette étude. Des simulations d'amarrage moléculaire et de dynamique moléculaire ont été menées pour évaluer les interactions protéine-protéine entre les protéines apoptotiques identifiées et imiter les voies apoptotiques intrinsèques des mammifères. Les résultats ont montré que les deux protéines pro-apoptotiques interagissaient avec la poche hydrophobe de l’orthologue anti-apoptotique, formant un complexe stable. Cette interaction peut représenter un événement critique dans une voie apoptotique chez *L. donovani*. Pour le caractériser davantage, nous avons utilisé des approches CRISPR-Cas9 pour cibler les protéines identifiées. Des mutants de population purement « knockout » et des cellules mutantes surexprimant l'épisome ont été exposés à des stimuli apoptotiques. Le test de marquage des extrémités de l'ADN par la désoxynucléotidyltransférase terminale dUTP (TUNEL) et le profil d'expression quantitatif suggèrent que ces protéines sont impliquées dans l'apoptose du parasite et pourraient jouer un rôle dans la survie du parasite.

## Introduction

Visceral leishmaniasis (VL) is a vector-borne chronic disease in children and elderly immunocompromised people. It is still a major tropical and/or sub-tropical neglected illness. Its prevalence is influenced by sand fly species traits, local ecosystem dynamics of transmission locations, current and prior parasite exposure to the human population, and human behaviour [14]. Since VL was first studied in India over a century ago, researchers have seen incidence cycles that climb and fall with some regularity. According to the World Health Organisation (WHO), an estimated 50 000 to 90 000 new VL cases are reported worldwide annually, of which 90% are recorded in three eco-epidemiological hotspots regions, including the following ten countries: Brazil, China, Ethiopia, Eritrea, India, Kenya, Somalia, South Sudan, Sudan, and Yemen [47]. The causative agent of VL is a protozoan parasite of the *Leishmania donovani* complex transmitted by a sand fly. In the Indian subcontinent, the species responsible for VL is *L. donovani*, with humans as the primary reservoir and *Phlebotomus argentipes* as the main vector [32]. In the Mediterranean basin, the Middle East, Central Asia, South America, and Central America, VL is caused by *L. infantum* and dogs are the principal reservoir of the parasite [10]. A number of chemotherapeutic options are available to control the spread of VL in the endemic zones. Chemotherapeutic agents [53] like pentavalent antimony, amphotericin B, miltefosine, paromomycin, pentamidine, and liposomal amphotericin B [48] are used over time with their advantages and disadvantages. Immunotherapy is a relatively new approach to modulating the host immune response with effective biological molecules. Even though immunotherapy constitutes a wide range of therapeutics and prophylactics, this approach is in its early stage of development as therapeutics against leishmaniasis. These currently available treatments elicit undesirable side effects, show administration limitations, and are costlier therapeutic interventions, and the advent of drug-resistant genotypes renders this approach ineffective [46]. These limitations in leishmaniasis are a severe worldwide health concern, especially in developing and endemic countries.

To address these constraints, efforts are constantly made to enhance the understanding of the aetiology and pathogenesis of leishmaniasis [50]. In this context, it is interesting to highlight that leishmanial parasites undergo regulated apoptotic-like cell death during their life cycle and pathogenesis, phenotypically similar to higher eukaryotic apoptosis [27]. *Leishmania,* inside the sand fly gut, manages the promastigote population, choosing infectious forms ideal for disease transmission. Promastigotes that do not mature into virulent metacyclic forms are terminated in a regulated manner to help the metacyclic forms by restricting the limited nutrition supply of the procyclic forms [16]. Such an apoptosis-like events, like eukaryotic apoptosis, help control parasite density within the host organism. This regulation prevents hyperparasitism, host mortality, and the transmission of non-infectious parasites [41]. In addition, macrophage phagocytising apoptotic *Leishmania* cells inhibit pro-inflammatory cytokine secretion and increase anti-inflammatory signals [4, 21]. One can infer that *Leishmania* has retained such apoptosis-like cell death mechanisms during evolution because they are advantageous or necessary for the survival of the species or populations [28, 51]. In contrast to higher eukaryotes, understanding apoptosis-like cell death mechanisms in *Leishmania* is still inadequate. This is because the molecular machinery required for initiating and executing apoptosis-like cell death is yet to be identified and defined. Furthermore, it is crucial to understand protein-protein interactions that necessitate cellular structure, function, and organisation [15]. Protein-protein interactions mediate most biological activities; hence, a complete description of the association process at the molecular level is required to understand the fundamental mechanisms. Molecular docking provides a tool for basic research by computationally predicting the interaction of two molecules. Protein-protein docking techniques, for example, seek to discover the natural binding mechanism between two proteins. In this study, we carried out a cross-search for orthologous genes through the genome of *L. donovani* against mammalian essential apoptotic Bcl-2 family proteins that include anti-apoptotic proteins (Bcl-2, Bcl-XL, Bcl-W, MCL-1, BFL-A1), pro-apoptotic pore formers (BAX, BAK, BOK), and pro-apoptotic BH3 only proteins (BAD, BID, BIK, BIM, BMF, HRK, NOXA, PUMA) [29]. Bcl-2 family proteins control and cause mitochondrial outer membrane permeabilisation, an essential process in apoptosis and an ideal candidate for orthologous gene search. Orthologous genes are one of the two fundamental homologous genes that arose through speciation from a single ancestral gene, hence representing the evolution of species [22]. All Bcl-2 family proteins have at least one conserved Bcl-2 homology (BH) domain that is required for significant apoptotic function and interactions. Based on its primary function, the Bcl-2 family is divided into three groups: (a) anti-apoptotic proteins, (b) pro-apoptotic pore-formers, and (c) pro-apoptotic BH3-only proteins [40]. We found orthologs of anti-apoptotic and pro-apoptotic proteins and docked them. Similarly, *L. donovani*'s BH3 domain-containing protein was also chosen and docked with anti-apoptotic orthologs. The stability of the docked complex was assessed using a molecular dynamics simulation study, which revealed a plausible apoptotic route involving an anti-apoptotic protein and a protein with a BH3 domain. In the second stage, we wanted to use reverse genetics to obtain experimental validation and demonstrate the role of the proteins identified by the bioinformatics analysis in the apoptotic process. Thus, overexpression of tagged proteins and CRISPR-Cas9-based knock-out were used. The generated mutant parasites were characterised by quantitative expression profiling in the presence and absence of an apoptotic stimulus. Our *in silico* and experimental findings suggest, for the first time, the significance of the identified protein in apoptosis and propose a putative interacting model for *Leishmania* apoptosis or apoptosis-like events.

## Materials and methods

### Search for apoptotic orthologs of *L. donovani*

Identification of orthologs may yield functional insights for genes with unknown functions and is an essential step in evolutionary reasoning [34]. Here, in-house OrthoMCL [38] from eukaryotic pathogen, vector, and Host informatics resources (VEuPathDB, https://veupathdb.org/veupathdb/app, last accessed 15 November 2024) was employed to identify orthologs using the Markov cluster approach [3] Afterwards, the MCL (Markov clustering algorithm) mega clustering tool differentiates each protein pair considering evolutionary distances.

### Structure modeling, refinement and validation of the *Leishmania* apoptotic ortholog proteins

To identify the *Leishmania* apoptotic proteins’ binding sites, residue modeling was performed, and three-dimensional structures were generated. The ortholog search results subsequently did not have the three-dimensional structure resolved to date. Therefore, structure and structure details about the protein’s binding site and binding residues are unavailable. Hence, for the *in silico* structural prediction of protein, the sequence of orthologs explored within the genome of *L. donovani’s* BPK strain in reference to mammals was retrieved from the VEuPathDB (https://veupathdb.org/veupathdb/app, last accessed 15 November 2024). The protein sequences were used for the 3D structure prediction by exploring the Phyre2 web-based server to predict protein structure [31]. The modern template-based modeling approach failed to produce realistic structures for less-conserved local regions even when the overall structure can be reliably predicted. Less-conserved or unreliable local regions (ULRs) are frequently associated with functional specificity, and *ab-initio* refinement is invaluable for further functional and design studies [45]. So, the predicted structures were further refined on the GalaxyWeb refined web-based server to attain its nearest native 3D conformation [33]. Furthermore, the quality of refined models was validated by analysing the QMEANDisCo [52] score and their structural conformations on the Ramachandran plot using the structure assessment tool of the SWISS-MODEL server (https://swissmodel.expasy.org/assess, last accessed 15 November 2024).

### Computational molecular study to investigate interactions between apoptotic proteins of *Leishmania*

Molecular docking generates critical structural information and interaction between protein-protein and/or protein-ligand molecules. The molecular docking algorithm ranks and picks potential hits based on their binding energy score [44]. ClusPro [35], HDOCK [55], and pyDock [26] online docking tools were employed for docking investigations which further generated ten best docking orientations, and the best docked orientation based on the lowest energy was selected for further studies. Molecular dynamics simulations are a powerful, advanced tool to understand molecular processes at a resolution that is often unattainable by experiment and explore atomic-level mechanisms that are often outside the purview of existing experiment methods [1]. Here, UAMS-Simlab WebGro Macromolecular simulation was used by employing the GROMACS simulation programme [20]. WebGro is a fully automated and easy-to-use computational biology tool WebGro for Macromolecular Simulations; (https://simlab.uams.edu, last accessed 15 November 2024). Here, molecular dynamics simulations were executed to investigate the stability of *Leishmania* pro-apoptotic and anti-apoptotic complexes in an explicit water solution at 300K temperature. The protein complex combinations were placed in a triclinic box with a GROMOS96 43a1 force field. By introducing NaCl counter-ion, the solvated system was neutralised. Solutes were treated to NVT/NPT ensemble to equilibrate the system.

### *Leishmania donovani* culture

Promastigote form of *L. donovani* strain 1S2D (MHOM/SD/62/1S-CL2D, referred to here as LD1S) was grown at 27 °C, in chemically defined HOMEN culture with 10% foetal calf serum (FCS) and 0.005% Hemin (3.5 mg/mL) [7]. The cell culture density at the start was 1 × 10^6^ cells/mL, as assessed by the CellDrop FL (DeNovix automated cell counter).

### Generation of *Leishmania donovani* T7-Cas9 expressing parasites

Plasmid pTB007 [6] for T7 RNA polymerase and Cas9 expression was transfected into LD1S to achieve LD1S-T7-Cas9-expressing cells. The plasmid also conferred hygromycin resistance, which was used to select transfected cells. In the Amaxa Nucelofactor IIb (Lonza, Basel, Switzerland) with 1X Tb-BSF transfection buffer, one pulse with programme X-001 was used for transfection.

### Primer design to generate *Leishmania* knock-out and quantitative PCR

*Leishmania* apoptotic genes target sequences were obtained from TriTrypDB (https://tritrypdb.org/tritrypdb/app, last accessed 15 November 2024) using their unique gene ID. Primers for amplifying donor DNA and sgRNA targeting genes of interest (GOIs) from pT, pPLOT, and pLPOT plasmids were designed using LeishGEdit (http://leishgedit.net/ last accessed 15 November 2024), as described by Beneke et al [6]. To ensure specificity, the primer sequences were checked against the complete genome using the TriTrypDB BLAST tool. Quantitative PCR primers were designed using Roche’s LC480 probe design software. The primer design used SYBR green with a Tm target of 60 and a size product smaller than 200 as parameters. IDT Belgium synthesised all designed primers (see Supplementary Table 1).

### Generation of mutants

Donor DNA with puromycin and gentamicin drug resistance was amplified by PCR using the pTPuro and pTNeo plasmid backbones. LD1S T7-Cas9 cells were transfected with PCR-amplified donor DNA for KO and sgRNA template targeting apoptotic genes. The concentration used per transfection of donor DNA and sgRNA used were in the range of 25–30 μg (two KO cassettes and two sgRNA templates). For overexpression and addback (AB) experiments, the apoptotic gene was cloned into a pTH6nGFPc vector [19] in frame with a GFP tag. The resulting plasmid was transfected into LD1S promastigotes. For all transfections, cells were in exponential phase with cells in the range of 5–6 × 10^6^ cells/mL.

### Miltefosine half maximal inhibitory concentration (IC_50_) determination

Miltefosine (MLT) stock of 20 mM was prepared in dH_2_O (SIGMA M5571-50 mg). To determine the IC_50_ of MLT, a cytotoxicity and viability test was performed using exponentially growing promastigotes at a concentration of 2 × 10^6^ cells/mL. The parasites were plated in 96-well plates and treated with miltefosine for 72 h, followed by treatment with methylthiazolyldiphenyl-tetrazolium bromide (MTT) for 4 h. MTT, a membrane-permeable yellow dye, is reduced by mitochondrial reductases in living cells to form purple formazan. The optical density was measured at two wavelengths: 570 nm, corresponding to the absorption peak of formazan, and 690 nm, a background absorbance far from the peak.

### TUNEL assay to investigate apoptosis

*In situ* detection of DNA fragments following treatment of *Leishmania* with MLT 5 μM and 10 μM for 24 h was measured by TUNEL using a Cell Death Detection kit (*In Situ* Cell Death Detection Kit, POD; Roche; Cat. No. 11 684 817 910). One mL of *Leishmania* cells from the 5 mL culture (with and without MLT; post 24 h treatment) was centrifuged (3 000 rpm for 5 min), resuspended in 1 mL phosphate-buffered saline (PBS) 1X, washed with PBS 1X, and fixed with 500 μL of 4% paraformaldehyde for 1 h after washing with PBS. 40 μL of fixed cells were deposed on one ThermoFisher Scientific Menzel-Glaser Superfrost Plus Gold slide per well and air dried. Wells were washed with PBS 1X and incubated with H_2_O_2_ (3% in methanol) for 10 min at 4 °C. They were then washed twice with PBS 1X, placed on ice and permeabilised with freshly prepared, chilled 0.1% sodium citrate in 0.1% Triton X-100 solution for 2 min. For labelling preparation, the positive control well was treated with DNAse (DNase I, EN0525 – 1 U/L; Invitrogen, Waltham, MA, USA) for double-strand break. A negative control cell was added with 50 μL of Vial 2 of TUNEL kit without terminal transferase, and 50 μL of TUNEL mixture containing terminal transferase was added per well, including the positive control well. Slides were incubated in a humidified chamber at 37 °C for 1 h and washed with PBS. Cells were then incubated with 30 μL of diluted Hoechst 33342 (Thermo Fisher Scientific, # H3570) for 5 min and rewashed with PBS 1X, and 7 μL of SlowFade^TM^ Gold antifade reagent (Invitrogen, # S36937) was added to each well. Slides were covered with a 1.5H cover slip.

### Mitotracker staining imaging and acquisition

Mid-log LdAQP-GFP cells at a density of approximately 3 × 10^6^ cells per mL were incubated with Mitotracker (Invitrogen/Molecular Probes, # M7512) at a final concentration of 100 nM for 15 min at 27 °C in culture medium without FCS. The culture was then washed three times and left for one additional hour of growth in a complete medium. Cells were washed twice in PBS 1X, and a small volume of concentrated cells was deposited on poly-lysin home-coated slides. Nuclear and kinetoplast DNAs were stained with Hoechst 33342 (Thermo Fisher Scientific, # H3570). ProLong™ Live antifade Reagent (Invitrogen, # P36975) and a 1.5H coverslip were used. All images were acquired using Zen software, on a Zeiss^®^ Axioimager Z1 microscope equipped with an ORCA-Flash 4 OLT CMOS camera (Hamamatsu^®^) with 63x objective (Plan-Apochromat 63x/1.40 Oil) and filters adapted for the different fluorescence used.

### RNA extraction and cDNA synthesis by reverse transcription

MLT post-24 h treatment cells were recovered by centrifugation at 3 000 rpm for 3 min, and total RNA was extracted using a RNeasy Mini Kit protocol (QIAGEN, # 74104). Extracted total RNA was treated with Turbo DNAse (Invitrogen^TM^, # AM1907) to obtain an RNA free from genomic DNA contamination. Post DNAse treatment, the extracted RNA was transcribed into cDNA using a kit (Invitrogen^TM^ SuperScript^TM^ III First-strand Synthesis Super Mix for qRT-PCR, # 11752050) containing Oligo dT with PolyA tails, enabling the reverse transcription of mRNAs. No RT treatment was used for the qPCR as a negative control.

### Primer pair and qPCR efficiency

Primer pairs for quantitative PCR were assessed on genomic DNA for PCR efficiency, reproducibility, and primer pair efficiency. Seven dilutions (D) of gDNA starting at 1 ng/μL concentration as D1 with 10-fold decreased concentration to D7 was used for primer pair efficiency and performed in triplicate for each primer pair mix. Primer pair mix with 7 μL of 2X SYBR green (LightCycler 480 SYBR Green I Master; Roche, # 04707516001) and 1 μL of 10 μM of forward and reverse primer was prepared for all gene targets, including the reference gene. Then, 5 μL of gDNA dilutions were added in triplicate, and 5 μL H_2_O as a negative control in triplicate was included on 96-well plates. The PCRs were performed on Roche Light Cycler 480 with run specifications: Tm = 60 °C, elongation time = 40 seconds with 45 cycles.

### Quantitative expression of *Leishmania* apoptotic genes in wild type (WT) and mutant strains

cDNA prepared from mRNA by First-strand synthesis Superscript III reverse transcriptase was amplified in a Roche LightCycler 480 Real-Time PCR machine using 2X SYBR green. The PCR conditions were 95 °C denaturation, followed by a combined annealing and elongation step at 58 °C. The data were analysed using the LightCycler 480 software against a standard curve obtained from serial gDNA dilutions. A cDNA concentration of 100 ng/μL was used in triplicate with water and no-RT-treated RNA as a negative control. Data from three independent experiments for WT and mutant cells were normalised to the cGAPDH (LdBPK_362480.1) as a reference gene.

### Statistical analysis

To statistically validate the change in expression (qRTPCR) and number of TUNEL positive cells and TUNEL negative cells in comparison to WT, One-Way Analysis of Variance (ANOVA) was performed. For the MLT-treated TUNEL assay, ANOVA was performed on log values converted from percentages. ANOVA calculation was performed in GraphPad Prism, and *p* values < 0.05 were deemed statistically significant. MS-Excel was used to plot all the graphs, and the asterisk “*” was used to present the significant *p* values.

## Results

### In silico screen to identify *L. donovani* proteins involved in an apoptotic pathway

#### Selection of four Bcl-2 family proteins in *L. donovani*

The Bcl-2 family proteins comprise both pro- and anti-apoptotic proteins. In this work, the OrthoMCL algorithm identified one ortholog linked with antiapoptotic function, LdBPK_302600.1, and two pro-apoptotic orthologs linked with pro-apoptotic function: LdBPK_291360.1 for pore formers and LdBPK_020680.1 pro-apoptotic BH3-only Bcl-2 family proteins. In TriTrypDB, LdBPK_302600.1 is annotated as “RNA-binding protein, putative”, LdBPK_291360.1 as “ATP-dependent Clp protease subunit, heat shock protein 100 (HSP100), putative”, and LdBPK_020680.1 as “ATP-dependent Clp protease subunit, heat shock protein 78 (HSP78), putative”. A putative AQP protein containing a pro-death BH3 domain and identified as gene Id LdBPK_221270.1 was also selected for its putative pro-apoptotic activity due to the BH3 domain and its possible interaction with anti-apoptotic orthologs [25]. The BH3 domain is required for the BCL-2 family member’s primary apoptotic function and intracellular membrane interactions [13].

#### Homology modeling of *Leishmania* apoptotic proteins

**Figure 1:**
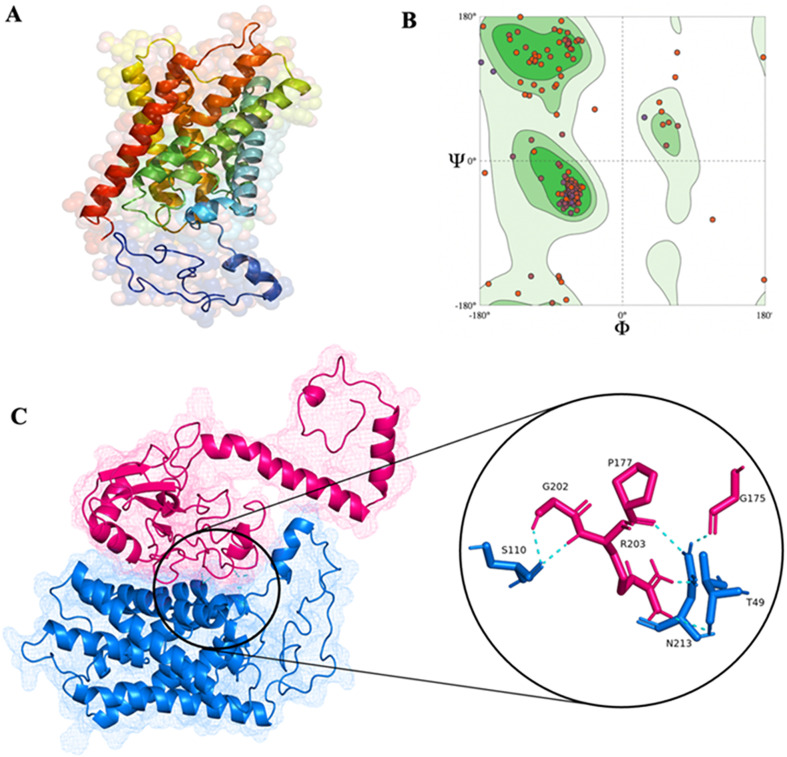
*In silico* structure and binding prediction of *Leishmania donovani* aquaporin (AQP). (A) Homology structure modeled by Phyre2 for AQP; (B) Ramachandran plot for ϕ and ψ angles for the validation of homology predicted AQP structure; (C) Docking prediction of AAPO in pink and AQP in blue with their interacting residue in the inset image.

*Leishmania* protein structures were generated using the homology modeling technique because they were unavailable in the protein data bank. The homology model of identified Bcl-2 family proteins and BH3 domain-containing AQP protein of *L. donovani* was produced using the Phyre2 homology modeling server for 3D structural characterisation (Fig. 1). Phyre2 modeled the proteins with 78% residue with more than 90% confidence for LdBPK_302600.1 (AAPO), 99% residue modeled with 90% confidence for LdBPK_291360.1 (PAPO-I), and 88% residue modeled with 90% confidence for LdBPK_020680.1 (PAPO-II) and LdBPK_221270.1 (AQP). AAPO and PAPO are acronyms used hereafter throughout the manuscript based on the Anti/Pro apoptotic putative role based on our *in silico* findings. The modeled protein was refined using the GalaxyWeb refine webserver and validated for final structure using the SWISS-MODEL structural assessment tool. According to the Ramachandran plot investigation, the most preferred and allowed locations for the statistical distribution of ϕ and ψ angles combination of amino acid residues were 91.92% for AAPO, 91.2% for PAPO-I, 92.27% for PAPO-II and 93.66% for AQP, indicating the high accuracy of the models (Figs. 1A–1B).

### Molecular docking studies to assess the interaction of anti-apoptotic with pro-apoptotic proteins

#### Identification of the pro-apoptotic and AQP binding sites on Bcl-2 anti-apoptotic protein of *L. donovani*

**Supplementary Figure 1:**
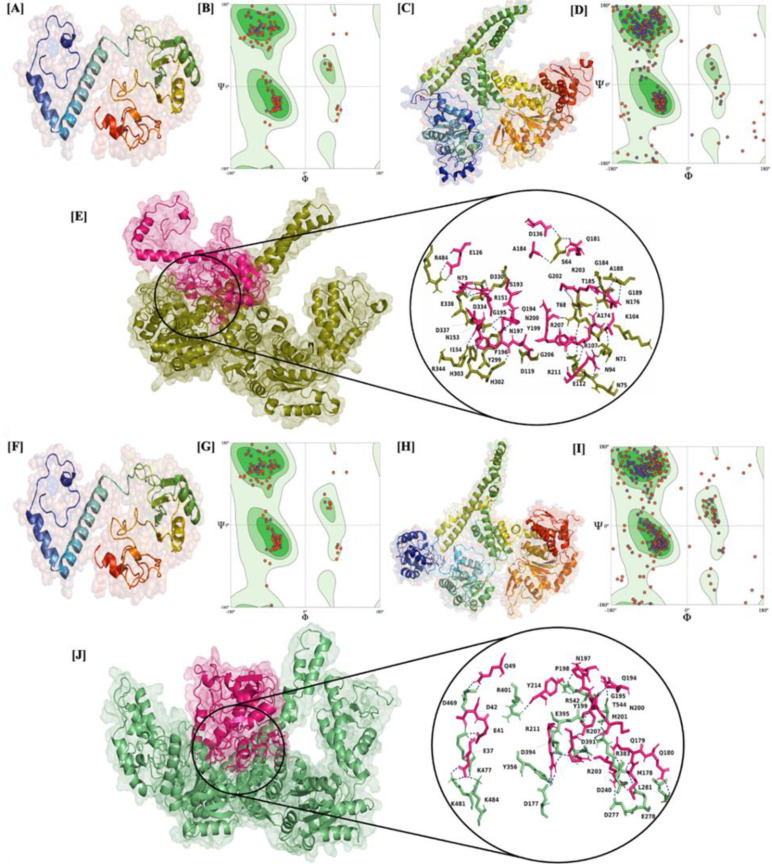
*In silico* structure and binding prediction of different orthologs.

Pro-apoptotic and anti-apoptotic proteins were randomly docked in two combinations, PAPO-I + AAPO (combination I) and PAPO-II + AAPO (combination II) (Supplementary Fig. 1). It was found that the docked score trends between these two combinations were comparably similar except for HDOCK (Table 1). The alignment and polar interaction residues for AAPO were similar in both combinations, with a higher docking score for combination II. In contrast to the previous combinations, AQP + AAPO (combination III) (Fig. 1C) showed superior binding energy (Table 2). Anti-apoptotic proteins in mammalian intrinsic pathways adopt a highly conserved tertiary structure, forming a hydrophobic BH3 domain-binding groove that behaves as a binding site for other BH3 domain proteins [36, 40]. The BH3 domain is required for primary apoptotic function and interplay with intracellular membrane proteins [49]. We did not find binding through the BH3 domain of AQP in mammals. We did, however, locate hydrophobic amino acids at AAPO protein-interacting sites. Combination III forms an encouraging hydrophobic domain groove through which the interaction occurs.

**Table 1:** Docking energies of combinations I (PAPO-I + AAPO) and II (PAPO-II + AAPO) with their interacting residues in kcal/mol.

Docking server		Docking complex	
		Combination I	Combination II
ClusPro	Energy	−1125.6 kcal/mol	−1176.5 kcal/mol
	Polar contacts on Pro-apoptotic	D177, D240, D277, E278, L281, Y356, R383, D391, D394, E395, R401, D469, K477, K481, K484, R542, T543	S64, T68, N71, N75, N94, K104, R107, D119, G184, T185, A188, G189, Y299, H302, H303, D330, D334, D337, E338, R344, R484
	Polar contacts on AAPO	E37, E41, D42, Q49, M178, Q179, Q180, Q194, G195, N197, P198, W199, N200, M201, R203, R207, R211, Y214	N75, E126, D136, R151, N153, I154, A174, N176, Q181, A184, S193, Q194, G195, F196, N197, Y199, N200, G202, R203, G206, R207, G210, R211
HDOCK	Energy	−305.75 kcal/mol	−279.25 kcal/mol
	Polar contacts on Pro-apoptotic	R18, E141, R142, D155, N156, A246, R257, Q358, R401, S405, Q472, E480, K627, S831	N75, P93, R117, E348
	Polar contacts on AAPO	E28, R34, S68, Q71, T72, T74, I102, T103, E124, E126, Q194, N197, P198, R207	R159, Y199, R203, R206
pyDock	Energy	−35.872 kcal/mol	−44.356 kcal/mol
	Polar contacts on Pro-apoptotic	R17, K25, E95, K259, N263, N296	R34, T55, R57, S229
	Polar contacts on AAPO	E37, N197, Y199, R203, Y214	D17, D22, D24, E28

**Table 2:** Docking energy of combination III (AQP + AAPO) with its interacting residue in kcal/mol.

Server	Docking complex	Combination III
ClusPro	Energy	−1448.9 kcal/mol
	Polar contacts on AQP	T49, S110, N213
	Polar contacts on AAPO	G175, P177, G202, R203
HDOCK	Energy	−61.634 kcal/mol
	Polar contacts on AQP	F102, S106, S110, N213
	Polar contacts on AAPO	Q182, I182, G202, R203
pyDock	Energy	−317 kcal/mol
	Polar contacts on AQP	S99, N213
	Polar contacts on AAPO	N197, R211

### Molecular dynamics simulation studies of docked complexes suggest a model for the stable interaction

#### Assessing protein and amino acid dynamics: a comparative root mean square deviation (RMSD) and root mean square fluctuation (RMSF) analysis

**Supplementary Figure 2:**
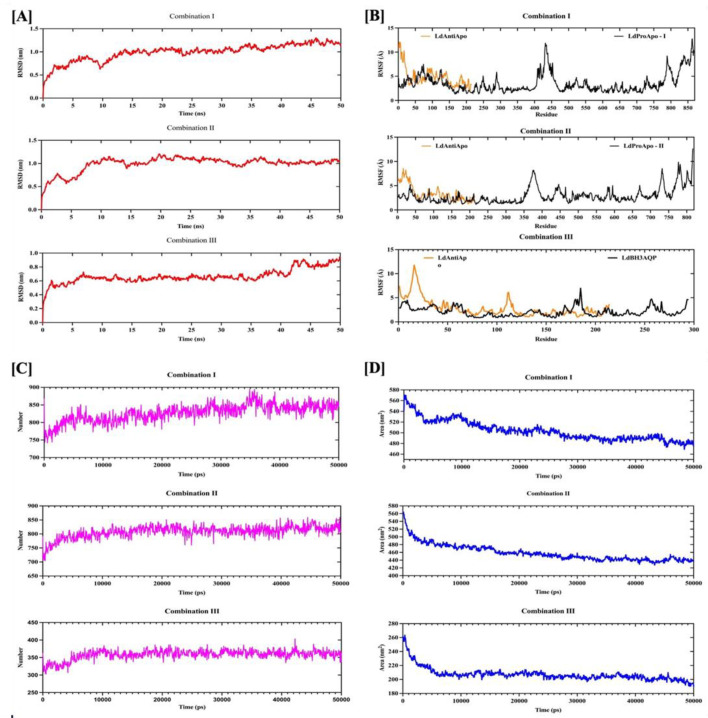
Trajectory graph evaluating dynamic behaviour and conformation consistency of the protein-protein complex over time.

Combinations I and II had average RMSDs of 0.98 and 0.81 nm, respectively, and combination III had an RMSD of 0.67 nm, showing that all complexes are coherent. As depicted (Supplementary Fig. 2A), the overall equilibrium for combination II was attained at 15 ns with an RMSD of 0.08 nm, whereas the equilibrium for combination III was at 5 ns with an RMSD of 0.06 nm. In the last 40^th^ ns of the simulation, a plateau graph was observed, which becomes stable shortly after 5 ns and continues below 1 nm for combination III, indicating that perhaps the system is steady and well equilibrated. Except for combination I, the RMSD computed for combinations II and III revealed no significant variations in the backbone, showing that the protein binding is stable and robust, with minimal conformational changes that do not affect the protein backbone stability during the simulation [17]. We explored the RMSF fluctuations of simulation studies to monitor the dynamic behaviour of amino acid residues during protein-protein interactions. The degree of chain flexibility in the three combinations was represented by the RMSF of each residue from its average time location (Supplementary Fig. 2B). Internal fluctuations, as well as conventional N- and C-terminal fluctuations, were found in all apoptotic proteins of *L. donovani*. Aside from the N- and C-terminal regions, the flexible area in the protein region, includes the residues that belong to the site linked explicitly to its protein partner interaction sites, as shown in Tables 1 and 2. Several other flexible residues were detected along with these and are thought to be allosteric protein sites because higher fluctuations or structural alterations occur in regions known to be critical for binding and/or catalysis.

#### Assessing protein compactness, solvent accessibility, and stability of protein complex

We plotted the radius of gyration (Rg) for different time frames in a dynamic system to evaluate the compactness of the structures as an indicator of the size and compactness of all combinations. Over the dynamics period, a stably folded protein maintains a reasonably constant Rg [39]. Based on the Rg plot (Supplementary Fig. 2C) showing protein compactness, we can conclude that among the three combinations, combination III had the lowest average Rg of 2.36 nm, while combinations I and II had an average Rg of 3.72 and 3.52 nm, respectively. Combination III had a relatively steady Rg after the 7^th^ ns throughout dynamics. The evident lowest and slightest variation Rg for combination III indicates that the protein is stable and compact, with the tightest protein packing. Solvent accessible surface area (SASA) is the surface area of a protein that evaluates the interaction of complexes with their solvent molecules [12]. Over 50 ns simulation, average SASA values for the combinations I, II, and III were recorded (Supplementary Fig. 2D) to be 503.46 nm^2^, 458.55 nm^2^, and 207.17 nm^2^, respectively. SASA values for combination I noticeably decreased with fluctuation during the simulation period; however, the SASA values of combination II and III did not fluctuate much and decreased over time, indicating structural compactness. The trajectory graphs of the apoptotic protein complex’s behaviour during the molecular dynamic simulation indicate that the protein-protein interaction of AAPO and AQP is far more stable than the other two combinations. The results revealed that the protein backbone of AQP with AAPO remained stable throughout the simulation period, with no notable fluctuations. AQP interacts with the hydrophobic surface AAPO to form a complex with a low binding score like mammalian anti and pro-apoptotic interactions. The backbone of LdPAPO-II, on the other hand, showed slight anomalous variations throughout the simulation period, whereas LdPAPO-I demonstrated instability with the AAPO.

### Experimental validation of *in silico* hits to explain *Leishmania* apoptosis

#### Exploring the impact of AQP gene expression on *Leishmania* apoptosis-like events

**Figure 2:**
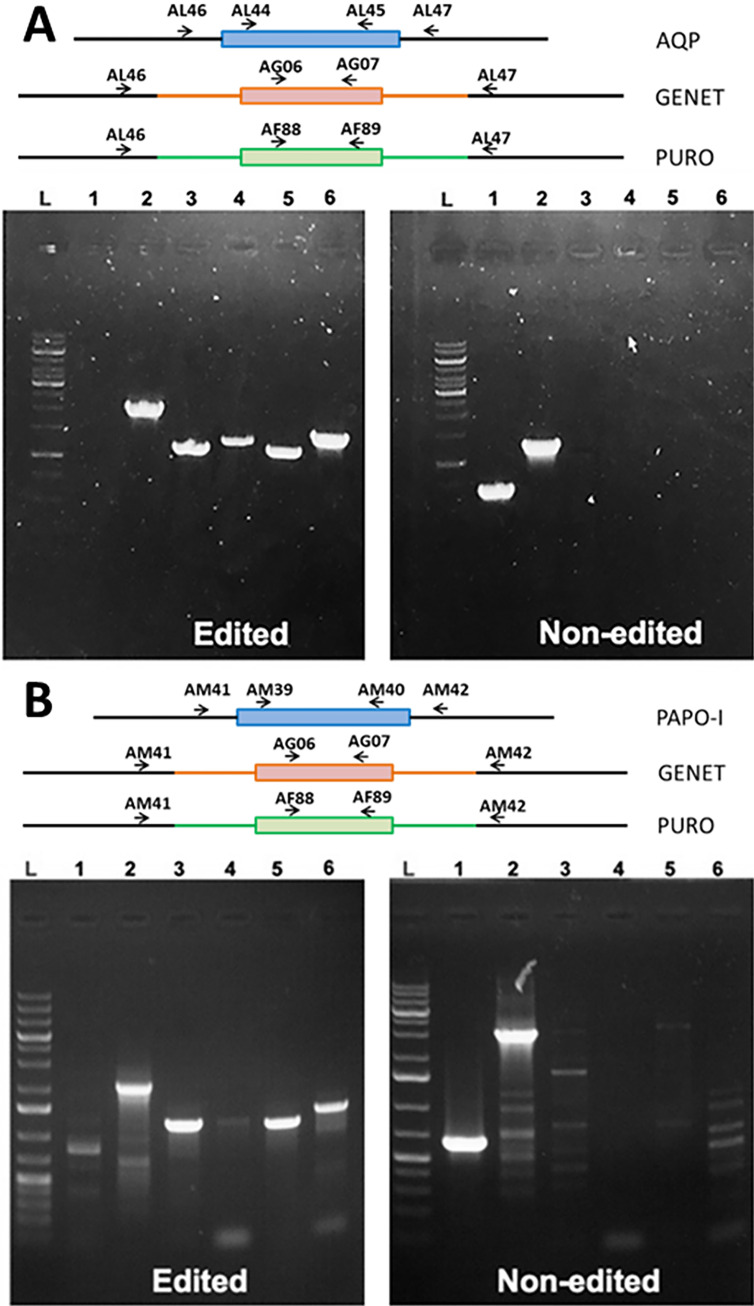
Validation of edition AQP and PAPO-I knock-out. Upper panels: schematic representation of each gene locus and primers (black arrows) used to confirm integration of the different drug resistant markers and loss of WT allele in the respective cell lines. Geneticin (GENET); puromycin (PURO). Wells: L is GeneRuler^TM^ 1 kb Plus DNA ladder, wells 1 to 6 represent the different primer pairs used to check for edition. [A] LdAQP KO; 1: AL44/AL45, no PCR amplification in edited and a positive band at 660 bp in non-edited cells, 2: AL46/AL47: Positive PCR amplification at either 1 959 bp (integration of geneticin resistance) or 2 056 bp (puromycin), or 1 239 bp (non-edited), 3: AL46/AG07: 1 137 bp (edited) and negative for non-edited, 4: AG06/AL47: 1 268 bp (edited) and negative for non-edited, 5: AL46/AF89: 1 114 bp (edited) and negative for non-edited, 6: AF88/AL47: 1 409 bp (edited) and negative for non-edited. [B] LdPAPO-I KO; 1: AM39/AM40, negative in edited and positive at 23 bp in non-edited, 2: AM41/AM42: Positive with 2 014/2 111 bp (edited – geneticin/puromycin) and 2 986 bp (non-edited), 3: AM41/AG07: 1 196 bp (edited) and negative for non-edited, 4: AG06/AM42: 1 263 bp (edited) and negative for non-edited; 5: AM41/AF89: 1 173 bp (edited) and negative for non-edited, 6: AF88/AM42: 1 405 bp (edited) and negative for non-edited.

The first protein we targeted was LdAQP, the BH3-domain containing putative AQP. We successfully performed a Cas9 KO (Fig. 2A), complementation (add-back) of the KO, and an episomal overexpression in the WT. This enabled us to look at the localisation of the protein, the changes in expression of the other target proteins as a function of AQP expression, and the percentage of TUNEL-positive cells under these different conditions.

**Figure 3:**
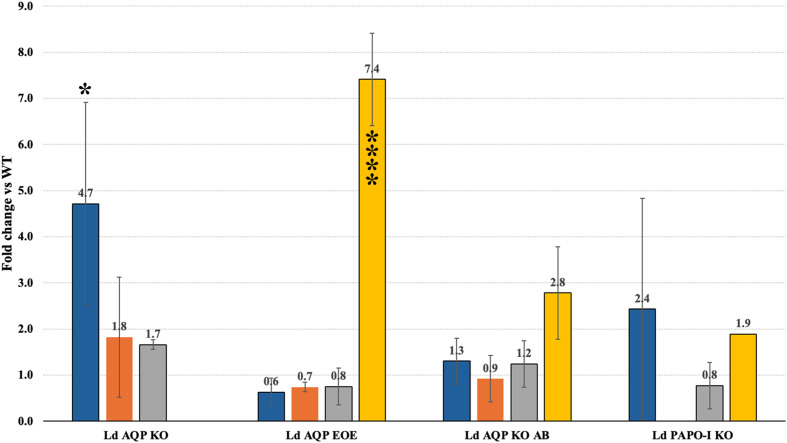
Bar graphs show the mean±sd fold change in gene expression when compared to the WT. All conditions were performed in triplicate in three independent experiments. Blue corresponds to AAPO; Orange to PAPO-I; Grey to PAPO-II; and Yellow to AQP. LD AQP KO: AQP knock-out; LD AQP EOEs: AQP episomal overexpression; LD AQP KO AB: AQP add-back; LD PAPO-I KO: PAPO-I knock-out. Statistically significant *p* < 0.005 (*p = 0.0388; ****p = <0.0001) are indicated.

**Figure 4:**
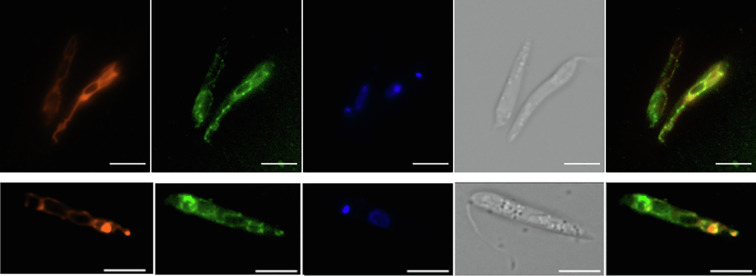
Localisation of LdAQP. From the left to the right, panels correspond to Mitotracker (red) staining, LdAQP-GFP (green) localisation, nuclear and kinetoplast DNA staining by Hoechst 33342 (blue), bright field and merged image of green and red fluorescence. Scale bar: 5 μm. A thin network typical of the mitochondrion was obtained in LdAQP-GFP (green) and Mitotracker (red) staining.

For episomal expression (add-back and overexpression), the LdAQP gene was cloned in a pTH6nGFPc vector [19] in a frame with a GFP tag and transfected into LD1S promastigotes. The quantitative expression of the AQP gene was checked and confirmed with qRT-PCR to confirm the overexpression of the AQP gene. qRT-PCR of the AQP presents an ~7-fold increase as compared to the WT LD1S cells (Fig. 3). The fluorescence due to the GFP tag also confirmed the presence of the AQP episomal expression (Fig. 4). GFP tag was used to determine the localisation of the protein in the *Leishmania*. Localisation of AQP-GFP was made up of a network and a few intense dots. Of note, an intense spot at the anterior part of the cell, close to the flagellar pocket was already noticed elsewhere [8]. To better define the localisation observation, the GFP-tagged AQP proteins were co-labelled with Mitotracker (Fig. 4). Co-localisation of the GFP and Mitotracker staining allowed us to determine that LdAQP localises to the mitochondrion. AQP KO parasites were obtained using a PCR-based CRISPR-Cas9 strategy [6]. We validated the KO by the targeted integration of the two resistance genes (puromycin and geneticin) and the non-amplification of the target gene by PCR (Fig. 2). This absence of gene-specific bands on the gel enabled us to verify a pure KO strain after cloning. Deletion of AQP was also checked by the absence of expression of the AQP gene with qRT-PCR (Fig. 3). After that, we performed other qRT-PCRs to investigate whether the absence of AQP modulates the expression of AAPO, PAPO-I, and PAPO-II developed in various AQP deleted mutant parasites. When AQP expression in KO parasites is zero, expression of AAPO significantly increased by 4.7 fold (*p*-value = 0.0388). An increase of around 2 fold was observed in the case of PAPO-I and PAPO-II in AQP KO mutants, but was not statistically significant. Overexpression of AQP by a factor of 7 tended to reduce the expression of the other three genes, but the change expression was non-significant (*p*-value = 0.9–0.8).

In the control complementation (in which a slight overexpression of AQP by a factor of 3 was observed), the expression of AAPO, PAPO-I, and PAPO-II was unchanged compared with the WT (Fig. 3). All changes in fold expression were statistically checked for significance with *p* < 0.05, which suggests that the expression of AAPO in LdAQP KO and AQP in Ld AQP EOE has significantly increased in comparison to the WT. These data favoured the hypothesis that these proteins form a complex, as suggested by the molecular docking studies.

**Figure 5:**
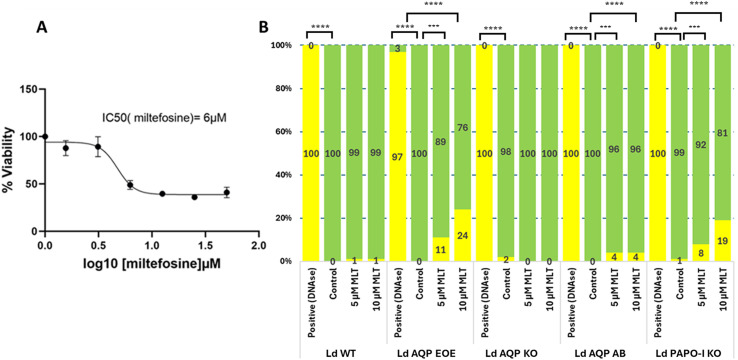
A. Determination of the IC50 of MLT. B. Apoptotic cells were detected using TUNEL assay. Histograms represent the percentage of Tunel positive cells (yellow) and Tunel negative cells (green). LD WT, LD AQP EOEs, LD AQP KO, LD AQP AB, and LD PAPO-I KO cells were treated with 5 and 10 μM MLT. DNase treated cells were used as positive control. Three experiments were performed, and 200 cells were counted by experiment. Statistically significant *p* < 0.005 (*p < 0.05; ***p < 0.0005; ****p < 0.0001) are indicated.

We then investigated whether AQP is implicated in increased sensitivity or resistance to an apoptotic stimulus. Among other inducers [5], we chose MLT to induce apoptosis. We used low doses of MLT, around the IC_50_ we determined at 6 μM (Fig. 5A). At 5 and 10 μM MLT, the TUNEL assay detected only ≤ 1% of apoptotic cells in WT and AQP KO cells (Fig. 5B). However, in the AQP episomal overexpressing parasites, the percentage of apoptotic cells increased to 11% and 24% in a dose-dependent manner (Fig. 5B). Of note, there is a slight increase of apoptotic cells in the AQP add-back parasite to 4%, which is probably linked with the slight overexpression (~3 fold) of AQP in that condition. These data suggested that AQP has a role in the apoptotic pathway in *Leishmania*.

#### Investigating the effects of identified apoptotic hit proteins on *Leishmania* apoptosis-like events

To assess the implications of our other identified apoptotic hit proteins, we attempted to knock out the other genes: AAPO, PAPO-I, and PAPO-II. We employed a similar PCR-based CRISPR-Cas9 KO approach and verification strategy to ensure the proper integration of the resistance marker and the complete absence of the target gene, as used for AQP. We were able to obtain true PAPO-I KO mutant parasites. The correct integration of the resistance marker and the complete absence of the PAPO-I was confirmed by PCR (Fig. 2B) and was further affirmed by the absence of expression by qRT-PCR (Fig. 3). As in LdAQP KO parasites, we also observed a shift in the quantitative expression profile of AAPO, PAPO-II, and AQP. AAPO and AQP were overexpressed around two-fold, and gene expression of PAPO-II was stable or decreased slightly by 0.8-fold of the value in the WT (Fig. 3). When we checked for sensitivity or resistance to apoptotic stimuli, PAPO-I KO was observed to be more sensitive to MLT; indeed PAPO-I KO cells presented dose-dependent sensitivity to MLT with TUNEL-positive cells at 8% and 19%, respectively, for the 5 and 10 μM concentrations compared to 1% in the absence of MLT and in the parental cells (Fig. 5). In two independent experiments for each locus, we were unable to generate true knock-outs of the AAPO and PAPO-II genes.

#### Analysing the quantitative expression of hit in MLT-treated mutant cells

**Figure 6:**
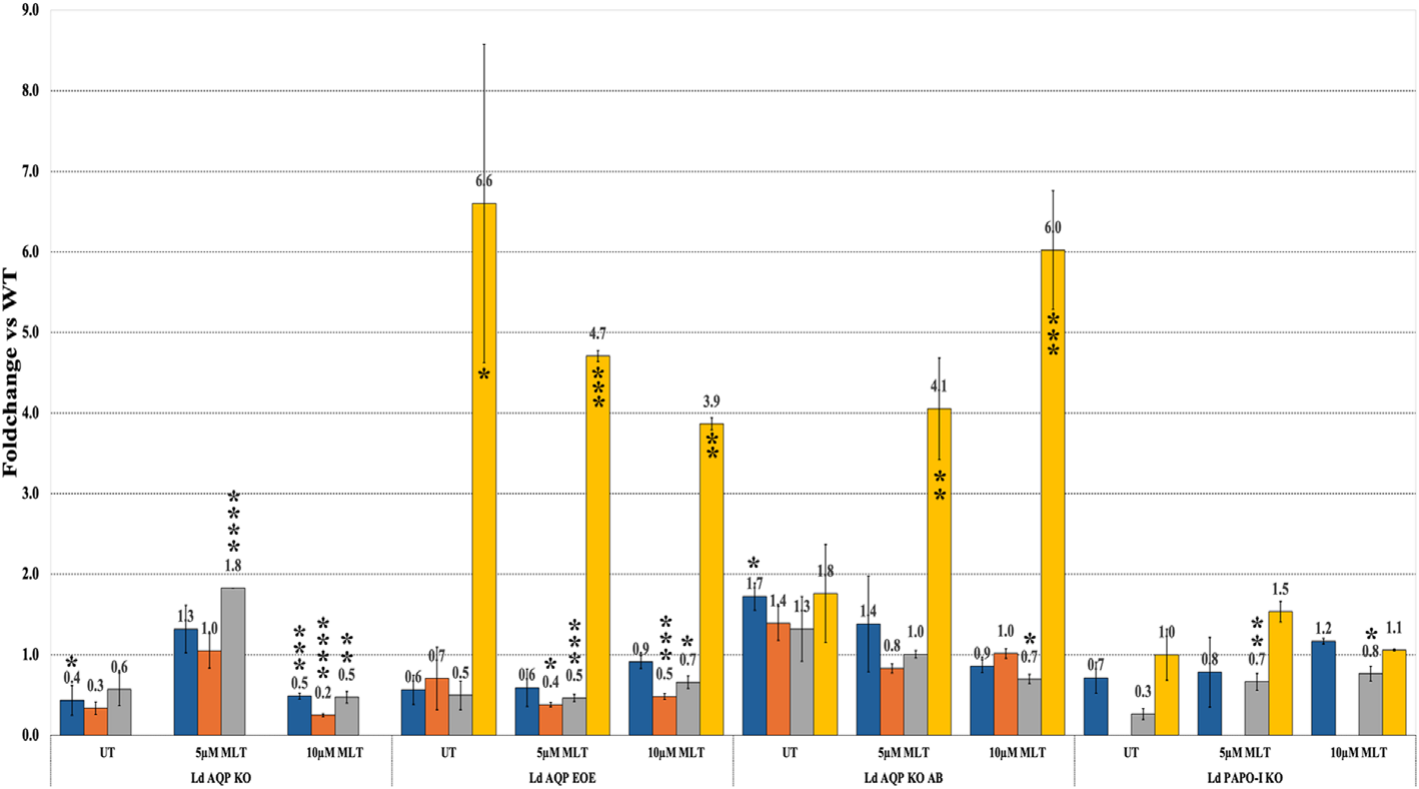
Fold change in AAPO in blue, PAPO-I in orange, PAPO-II in grey and AQP in yellow. The mutants LD AQP EOEs, LD AQP KO AB, LD PAPO-I KO when treated with varying concentration of MLT in comparison to untreated cells. All conditions were performed in triplicate. Statistical significant *p* < 0.005 (*p < 0.05; **p < 0.005; ***p < 0.0005; ****p < 0.0001) are indicated.

We examined the quantitative gene expression of AAPO, PAPO-I, PAPO-II, and AQP in MLT-treated mutant cells compared to WT. Mutant cells with varying concentration treatments were compared with treated WT cells, and untreated mutants were compared with untreated mutants, considered as controls. The quantitative expressions by qRT-PCR for genes were normalised again with cGAPDH. The fold change in mutant cells represents the expression profile compared to the WT (Fig. 6). These changes in the expression of hits were checked for statistical validation with calculated *p*-values below 0.05 (*p*-values with an asterisk “*”). AQP expression consistently decreased and increased dose-dependently for Ld AQP EOE and Ld AQP KO AB mutants, respectively, from 5 μM MLT (Ld AQP EOE, *p* = 0.0008; Ld AQP KO AB, *p* = 0.0016) to 10 μM MLT treatment (Ld AQP EOE, *p* = 0.0034; Ld AQP KO AB, *p* = 0.0004). While in Ld PAPO-I KO, AQP expression increased at first with 5 μM MLT treatment compared to WT and untreated cells, and then decreased with 10 μM MLT treatment, which was not statistically significantly validated. AAPO expression decreased significantly for Ld AQP KO in 10 μM MLT treatment (*p* = 0.0008), but no significant changes were observed in mutant cells. PAPO-I expression significantly decreased in 10 M MLT treatment (*p* < 0.0001) for Ld AQP KO, and in the case of Ld AQP, EOE expression decreased with 5 MLT (*p* = 0.0114) and increased in 10 M MLT treatment (*p* = 0.0004). At the same time, no significant changes were observed in Ld AQP KO AB. PAPO-II expression increased first for 5 MLT treatments (*p* < 0.0001) and then decreased for 10 MLT treatments (*p* = 0.0016) in Ld AQP KO mutant and consistently increased dose-dependently for Ld AQP EOE and Ld PAPO-I KO mutants, respectively, from 5 (Ld AQP EOE, *p* = 0.0008; Ld PAPO-I KO, *p* = 0.0073) to 10 μM MLT treatment (Ld AQP EOE, *p* = 0.0108; Ld PAPO-I KO, *p* = 0.0494), while significantly decreased in 10 MLT treated Ld AQP KO AB (*p* = 0.0194).

## Discussion

Apoptosis in higher eukaryotes, such as mammals, is a well-studied process with extensive knowledge about its molecular mechanisms, regulation, and physiological significance in development, tissue homeostasis, and disease. In contrast, apoptosis in unicellular divergent eukaryotes, particularly in organisms like *L. donovani*, ensures and controls the promastigote population in a limited nutrition environment inside the sand fly gut by selecting infectious forms and controls hyperparasitism and host mortality within the host that could lead to parasite transmission inhibition. The apoptosis-like events in *L. donovani* that support life form transition and pathogenesis should be studied further and better understood. We searched for evolutionarily conserved proteins and validated their role in an apoptosis-like process in *L. donovani*.

### CRISPR-Cas9 gene edition in *L. donovani*

As CRISPR-Cas9 has become the state-of-the-art strategy for editing genomes in general and the highly flexible *Leishmania* genome in particular (reviewed in [54]), we decided to use it in this study. We successfully adapted the CRISPR-Cas9 PCR-based strategy developed in *L. mexicana* [6] to *L. donovani.* Using this method, we succeeded in editing LdAQP and LdPAPO-I and obtaining null knock-out strains. However, we attempted to knock out the AAPO gene without success, suggesting its essential role in *Leishmania* promastigotes. This finding aligns with previous research indicating the essentiality of its ortholog in *T. brucei* (Tb927.6.3870) in procyclics, as determined by high-throughput phenotyping using RNAi target sequencing (RIT-seq) [2]. We also failed to obtain a true KO of PAPO-II, and like for AAPO, the locus was editable, but despite cloning, a WT copy of the targeted gene persisted, despite the correct integration of two selective markers. This is likely attributed to the flexibility of the *Leishmania* genome and the presence of mosaic aneuploidy, which contribute to the difficulty in obtaining stable knock-out mutants, even when multiple selective markers are employed [42,54]. Several attempts to remove all copies of PAPO-II were unsuccessful, including a knock-in strategy. The situation here is complicated by the presence of two copies of the gene at two loci, one on chromosome 2 and the other on 27, and the fact that the latter is not annotated on the LdBPK genome. Of note, two copies are present in the genome of most sequenced *Leishmania* species (LdCL_020012400-t42_1 and LdCL_270033400-t42_1, LINF_020012400-T1 and LINF_270033830-T1, LmjF.02.0710:mRNA and LmjF.27.2630:mRNA) and also in *T. brucei* (Tb927.2.3030 and Tb927.2.5980). A knock-in strategy supposedly not dependent on genome misannotation also failed. This suggests that this duplicated gene is essential for *Leishmania* promastigote growth. In the high-throughput phenotyping using RNAi target sequencing (RIT-seq) data, Tb927.2.3030 and Tb927.2.5980, which have non-homologous nucleotide sequences, are not essential genes, but this could be because they are functionally redundant.

### Tentative model for apoptosis in *L. donovani*

Combining our *in silico* and experimental findings, our screened proteins AAPO, PAPO-I, PAPO-II, and AQP are a few stakeholders of *Leishmania*’s apoptosis or apoptosis-like events. We have demonstrated that the response to MLT treatment differs between WT parasites, AQP deletion mutants, and AQP add-back mutants. The deletion mutants exhibit resistance to MLT, while overexpression of AQP renders the parasites sensitive to MLT. These findings indicate that AQP could act as a pro-apoptotic protein. One could infer that AQP is pro-apoptotic because it possesses the BH3 domain. This BH3 domain of Bcl-2 family proteins in mammals is responsible for binding mammalian pro-apoptotic pore former Bcl-2 proteins. The fine network observed with the GFP was highly suggestive of the single mitochondrion that characterise these parasites; the co-labelling with Mitotracker confirmed the mitochondrial localisation of LdAQP (Fig. 4). Additionally, in BLAST analysis, the highest score obtained for LdBPK_221270.1 (LdAQP) matches human Aquaporin 8, which is known to be a mitochondrial aquaporin, further supporting the mitochondrial localisation of LdAQP. Combining and comparing the TUNEL results and quantitative expression profile of MLT treatment on mutant cells, we noticed that the absence of AQP (in Ld AQP KO cells) rendered cells resistant to apoptotic stimuli. In the case of Ld AQP EOE and Ld AQP KO AB mutants, the expression of AQP is decreased and increased, respectively, in sync with an increased MLT concentration. The shift in AQP expression profiles makes cells sensitive to apoptotic stimuli. This AQP in higher eukaryotes belongs to the family of transmembrane water channel proteins required for water homeostasis. In addition to their primary function as water channels in mammals, they are essential for transporting glycerol, small solutes, and molecules, including carbon dioxide, metalloids, nitric oxide, ammonia, urea, and various ions [23]. Loss of cell volume or apoptotic shrinkage is an essential process in the cells entering the apoptotic pathway. AQPs, being influential in affecting the rate of water movement across the cell membrane, have been associated with the apoptotic volume decrease [9] and subsequent apoptosis. In a study in ovarian granulosa, the authors showed that inhibiting AQPs blocked apoptotic volume decrease and apoptotic events, such as cell shrinkage, changes in the mitochondrial membrane potential, DNA degradation, and caspase-3 activation, and, when overexpressed, showed loss of water permeability and higher apoptotic induction. Another study showed that overexpression of AQP confers resistance to apoptosis [24]. This conflicting finding suggests the possible role of AQPs in facilitating apoptotic volume decrease necessary for apoptosis. Another exciting and evident finding about aquaporins in *T. cruzi* is its presence in the acidocalcisomes and contractile vacuole, suggesting its role in the osmotic adaptions of parasites [43]. Acidocalcisomes are the compartments supposed to sequester *L. donovani* metacaspases [37] in their acidic environment and are synthesised in active form and released during the parasite apoptotic event. Our findings with AQP point to its role in *L. donovani* apoptosis, which is a big step forward in the molecular characterisation of apoptotic pathways in *Leishmania*. However, the relevance of AQP in osmoregulation, parasite homeostasis, and the function of the BH3 domain remains unknown.

According to our preliminary data on PAPO-I, it should rather be considered an anti-apoptotic protein, whereas our *in silico* search based on mammalian pro-apoptotic Bcl-2 protein is supposed to be pro-apoptotic. PAPO-I knock-out cells were sensitive to MLT, which is evident from the shift in expression profile in Ld AQP KO and Ld AQO EOE. Further complementation and localisation of PAPO-I are required to conclude that it is a pro or anti-apoptotic protein. Variation in the quantitative expression profile of all the identified proteins in mutants in the presence or absence of MLT as apoptotic stimuli suggests that they somehow interact and are important during *Leishmania*’s apoptosis or apoptosis-like events. However, more experiments are needed to finely describe the mutant parasite’s cellular defects and *in vitro* interacting partners. Fold change in candidate protein expression in mutants and WT cells in the presence and absence of apoptotic stimuli is promising. Still, this does not effectively correlate with a change in protein expression levels. For perspective, a quantitative proteomics study could shed more light on its expression change at the protein level, and a GFP-tagged pulldown study could explain the interacting partner in detail.

### A novel approach to therapeutic target discovery

The chemotherapeutic approaches employed for treating leishmaniasis have been associated with numerous side effects and the development of resistance. Immunotherapy and immunochemotherapy are newer therapeutic approaches gaining popularity, but they are still in their infancy. Understanding the pathogenesis and the pathogen’s metabolic pathways has led to the creation of several medications that target distinct biochemical pathways. We found encouraging findings for apoptotic proteins in *L. donovani* in this study and a potential apoptotic pathway. Our data suggest that AAPO and the duplicated PAPO-I gene are essential, making them targets of choice for therapeutic interventions. This was accomplished using orthologous gene screening in *L. donovani* versus key mammalian Bcl-2 apoptotic proteins. Molecular docking was used to investigate their interaction, and molecular dynamics simulation studies validated the interaction. These research findings clearly suggest unequivocally that the protein-protein complex combination III is a potentially crucial phase in the apoptotic process of *L. donovani*. Apoptosis [11] and BCL-2 family proteins [30] have been established and targeted as effective anticancer strategies owing to the dysregulation of pro-apoptotic Bcl-2 protein expression and the aberration of apoptotic signalling pathways that promote tumorigenesis. Drugs like venetoclax, which focuses on intrinsic apoptotic pathways, are already in use, and BH3-mimetics that directly provoke apoptosis by inhibiting pro-survival BCL-2 proteins are now in clinical testing as cancer therapies. Targeting apoptotic pathways and the Bcl-2 ortholog in *Leishmania* may provide effective anti-leishmanial therapies, as shown by the successful use of miltefosine, an anticancer drug, known to induce apoptosis-like cell death in *Leishmania* sp., in treating leishmaniasis [18].

## Conclusion

This is a first-of-its-kind work that identifies apoptotic proteins and pathways in *L. donovani* and presents a novel therapeutic target. We feel that further confirmation of the suggested protein-protein interaction would need experimental validation. Furthermore, the technique developed here yielded remarkable findings and may be used simultaneously to predict and uncover apoptotic protein orthologs in other diseases, as well as to foster multidisciplinary partnerships of expertise from experimental and computational scientists.

## Supplementary materials

The supplementary material for this article can be found at https://www.parasite-journal.org/10.1051/parasite/2024081/olm. *Supplementary Table 1*: Primers included in the study.

## References

[1] Adcock SA, McCammon JA. 2006. Molecular dynamics: Survey of methods for simulating the activity of proteins. Chemical Reviews, 106, 1589–1615. 16683746 10.1021/cr040426m PMC2547409

[2] Alsford S, Turner DJ, Obado SO, Sanchez-Flores A, Glover L, Berriman M, Hertz-Fowler C, Horn D. 2011. High-throughput phenotyping using parallel sequencing of RNA interference targets in the African trypanosome. Genome Research, 21(6), 915–924. 21363968 10.1101/gr.115089.110 PMC3106324

[3] Amos B, Aurrecoechea C, Barba M, Barreto A, Basenko EY, Bażant W, Belnap R, Blevins AS, Böhme U, Brestelli J, Brunk BP, Caddick M, Callan D, Campbell L, Christensen MB, Christophides GK, Crouch K, Davis K, DeBarry J, Doherty R, Duan Y, Dunn M, Falke D, Fisher S, Flicek P, Fox B, Gajria B, Giraldo-Calderón GI, Harb OS, Harper E, Hertz-Fowler C, Hickman MJ, Howington C, Hu S, Humphrey J, Iodice J, Jones A, Judkins J, Kelly SA, Kissinger JC, Kwon DK, Lamoureux K, Lawson D, Li W, Lies K, Lodha D, Long J, MacCallum RM, Maslen G, McDowell MA, Nabrzyski J, Roos DS, Rund SSC, Schulman SW, Shanmugasundram A, Sitnik V, Spruill D, Starns D, Stoeckert CJ, Tomko SS, Wang H, Warrenfeltz S, Wieck R, Wilkinson PA, Xu L, Zheng J. 2022. VEuPathDB: the eukaryotic pathogen, vector and host bioinformatics resource center. Nucleic Acids Research, 50, D898–D911. 34718728 10.1093/nar/gkab929 PMC8728164

[4] Arnoult D, Akarid K, Grodet A, Petit PX, Estaquier J, Ameisen JC. 2002. On the evolution of programmed cell death: apoptosis of the unicellular eukaryote *Leishmania major* involves cysteine proteinase activation and mitochondrion permeabilization. Cell Death & Differentiation, 9, 1, 65–81. 11803375 10.1038/sj.cdd.4400951

[5] Basmaciyan L, Casanova M. 2019. Cell death in *Leishmania*, Parasite, 26, 71. 31825305 10.1051/parasite/2019071 PMC6905399

[6] Beneke T, Madden R, Makin L, Valli J, Sunter J, Gluenz E. 2017. A CRISPR Cas9 high-throughput genome editing toolkit for kinetoplastids. Royal Society Open Science, 4(5), 170095. 28573017 10.1098/rsos.170095 PMC5451818

[7] Berens RL, Brun R, Krassner SM. 1976. A simple monophasic medium for axenic culture of hemoflagellates. Journal of Parasitology, 62(3), 360–365. 778371

[8] Biyani N, Mandal S, Seth C, Saint M, Natarajan K, Ghosh I, Madhubala R. 2011. Characterization of *Leishmania donovani* aquaporins shows presence of subcellular aquaporins similar to tonoplast intrinsic proteins of plants. PLoS One, 6(9), e24820. 21969862 10.1371/journal.pone.0024820 PMC3182166

[9] Bortner CD, Cidlowski JA. 2004. The role of apoptotic volume decrease and ionic homeostasis in the activation and repression of apoptosis. Pflügers Archiv 448, 313–318. 15107996 10.1007/s00424-004-1266-5

[10] Burza S, Croft SL Boelaert M. 2018. Leishmaniasis. Lancet, 392, 951–970. 30126638 10.1016/S0140-6736(18)31204-2

[11] Carneiro BA, El-Deiry WS. 2020. Targeting apoptosis in cancer therapy. Nature Reviews Clinical Oncology, 17, 395–417. 10.1038/s41571-020-0341-y PMC8211386 32203277

[12] Connolly ML. 1983. Solvent-accessible surfaces of proteins and nucleic acids. Science, 221, 709–713. 6879170 10.1126/science.6879170

[13] Cory S, Adams JM. 2002. The Bcl2 family: regulators of the cellular life-or-death switch. Nature Reviews Cancer, 2(9), 647–656. 12209154 10.1038/nrc883

[14] Courtenay O, Peters NC, Rogers ME, Bern C. 2017. Combining epidemiology with basic biology of sand flies, parasites, and hosts to inform leishmaniasis transmission dynamics and control. PLoS Pathogens, 13(10), e1006571. 29049371 10.1371/journal.ppat.1006571 PMC5648254

[15] De Las Rivas J, Fontanillo C. 2012. Protein-protein interaction networks: unraveling the wiring of molecular machines within the cell. Briefings in Functional Genomics, 11, 489–496. 22908212 10.1093/bfgp/els036

[16] Debrabant A, Nakhasi H. 2003. Programmed cell death in trypanosomatids: is it an altruistic mechanism for survival of the fittest? Kinetoplastid Biology and Disease, 2, 1–2. 12848897 10.1186/1475-9292-2-7 PMC194864

[17] Dong YW, Liao ML, Meng XL, Somero GN. 2018. Structural flexibility and protein adaptation to temperature: molecular dynamics analysis of malate dehydrogenases of marine molluscs. Proceedings of the National Academy of Sciences, 115(6), 1274–1279. 10.1073/pnas.1718910115 PMC5819447 29358381

[18] Dorlo TPC, Balasegaram M, Beijnen JH, De Vries PJ. 2012. Miltefosine: a review of its pharmacology and therapeutic efficacy in the treatment of leishmaniasis. Journal of Antimicrobial Chemotherapy, 67, 2576–2597. 22833634 10.1093/jac/dks275

[19] Dubessay P, Blaineau C, Bastien P, Tasse L, Van Dijk J, Crobu L, Pagès M. 2006. Cell cycle‐dependent expression regulation by the proteasome pathway and characterization of the nuclear targeting signal of a *Leishmania major* Kin‐13 kinesin. Molecular Microbiology, 59(4), 1162–1174. 16430691 10.1111/j.1365-2958.2005.05013.x

[20] Durrant JD, McCammon JA. 2011. Molecular dynamics simulations and drug discovery. BMC Biology, 9, 1–9. 22035460 10.1186/1741-7007-9-71 PMC3203851

[21] Fadok VA, Bratton DL, Konowal A, Freed PW, Westcott JY, Henson PM. 1998. Macrophages that have ingested apoptotic cells *in vitro* inhibit proinflammatory cytokine production through autocrine/paracrine mechanisms involving TGF-beta, PGE2, and PAF. Journal of Clinical Investigation, 101, 4, 890–898. 9466984 10.1172/JCI1112 PMC508637

[22] Fitch WM. 1970. Distinguishing homologous from analogous proteins. Systematic Zoology, 19(2), 99–113. 5449325

[23] Galán-Cobo A, Ramírez-Lorca R, Echevarría M. 2016. Role of aquaporins in cell proliferation: what else beyond water permeability? Channels, 10(3), 185–201. 26752515 10.1080/19336950.2016.1139250 PMC4954585

[24] Galán-Cobo A, Ramírez-Lorca R, Serna A, Echevarría M. 2015. Overexpression of AQP3 modifies the cell cycle and the proliferation rate of mammalian cells in culture. PLoS One, 10(9), e0137692. 26367709 10.1371/journal.pone.0137692 PMC4569366

[25] Genes CM, De Lucio H, Sánchez-Murcia PA, Gago F, Jiménez-Ruiz A. 2016. Pro-death activity of a BH3 domain in an aquaporin from the protozoan parasite *Leishmania**.* Cell Death & Disease, 7(7), e2318. 27468694 10.1038/cddis.2016.229 PMC4973364

[26] Jiménez-García B, Pons C, Fernández-Recio J. 2013. pyDockWEB: a web server for rigid-body protein–protein docking using electrostatics and desolvation scoring, Bioinformatics, 29(13), 1698–1699. 23661696 10.1093/bioinformatics/btt262

[27] Jiménez-Ruiz A, Alzate JF, MacLeod ET, Lüder CG, Fasel N, Hurd H. 2010. Apoptotic markers in protozoan parasites. Parasites & Vectors, 3, 104. 21062457 10.1186/1756-3305-3-104 PMC2993696

[28] Kaczanowski S, Sajid M, Reece SE. 2011. Evolution of apoptosis-like programmed cell death in unicellular protozoan parasites. Parasites & Vectors, 4, 44. 21439063 10.1186/1756-3305-4-44 PMC3077326

[29] Kale J, Osterlund EJ, Andrews DW. 2018. BCL-2 family proteins: changing partners in the dance towards death. Cell Death & Differentiation, 25(1), 65–80. 29149100 10.1038/cdd.2017.186 PMC5729540

[30] Kaloni D, Diepstraten ST, Strasser A, Kelly GL. 2023. BCL-2 protein family: attractive targets for cancer therapy. Apoptosis, 28, 20–38. 36342579 10.1007/s10495-022-01780-7 PMC9950219

[31] Kelley LA, Mezulis S, Yates CM, Wass MN, Sternberg MJ. 2015. The Phyre2 web portal for protein modeling, prediction and analysis. Nature Protocols, 10(6), 845–858. 25950237 10.1038/nprot.2015.053 PMC5298202

[32] Killick‐Kendrick R. 1990. Phlebotomine vectors of the leishmaniases: a review. Medical and Veterinary Entomology, 4, 1, 1–24. 2132963 10.1111/j.1365-2915.1990.tb00255.x

[33] Ko J, Park H, Heo L, Seok C. 2012.GalaxyWEB server for protein structure prediction and refinement. Nucleic Acids Research, 40, W294–W297. 22649060 10.1093/nar/gks493 PMC3394311

[34] Koonin EV. 2005. Orthologs, paralogs, and evolutionary genomics. Annual Review of Genetics, 39(1), 309–338. 10.1146/annurev.genet.39.073003.114725 16285863

[35] Kozakov D, Hall DR, Xia B, Porter KA, Padhorny D, Yueh C, Beglov D, Vajda S. 2017. The ClusPro web server for protein–protein docking. Nature Protocols, 12(2), 255–278. 28079879 10.1038/nprot.2016.169 PMC5540229

[36] Ku B, Liang C, Jung JU, Oh BH. 2011. Evidence that inhibition of BAX activation by BCL-2 involves its tight and preferential interaction with the BH3 domain of BAX. Cell Research, 21(4), 627–641. 21060336 10.1038/cr.2010.149 PMC3343310

[37] Lee N, Gannavaram S, Selvapandiyan A, Debrabant A. 2007. Characterization of metacaspases with trypsin-like activity and their putative role in programmed cell death in the protozoan parasite Leishmania. Eukaryotic Cell, 6(10), 1745–1757. 17715367 10.1128/EC.00123-07 PMC2043384

[38] Li L, Stoeckert CJ, Roos DS. 2003. OrthoMCL: identification of ortholog groups for eukaryotic genomes. Genome Research, 13(9), 2178–2189. 12952885 10.1101/gr.1224503 PMC403725

[39] Lobanov MY, Bogatyreva NS, Galzitskaya OV. 2008. Radius of gyration as an indicator of protein structure compactness. Molecular Bioliogy, 42, 623–628. 18856071

[40] Lomonosova E, Chinnadurai G. 2008. BH3-only proteins in apoptosis and beyond: an overview. Oncogene, 27(1), S2–S19. 10.1038/onc.2009.39 PMC2928556 19641503

[41] Lüder CG, Campos-Salinas J, Gonzalez-Rey E, van Zandbergen G. 2010. Impact of protozoan cell death on parasite-host interactions and pathogenesis. Parasites & Vectors, 3, 116. 21126352 10.1186/1756-3305-3-116 PMC3003647

[42] Martínez-Calvillo S, Stuart K, Myler PJ. 2005. Ploidy changes associated with disruption of two adjacent genes on *Leishmania major* chromosome 1. International Journal for Parasitology, 35(4), 419–429. 15777918 10.1016/j.ijpara.2004.12.014

[43] Moreno SN, Docampo R. 2009. The role of acidocalcisomes in parasitic protists 1. Journal of Eukaryotic Microbiology, 56(3), 208–213. 19527347 10.1111/j.1550-7408.2009.00404.x PMC2802266

[44] Morris GM, Lim-Wilby M. 2008. Molecular docking. Molecular Modeling of Proteins, 443, 365–382. 10.1007/978-1-59745-177-2_19 18446297

[45] Park H, Seok C. 2012. Refinement of unreliable local regions in template‐based protein models. Proteins: Structure. Function and Bioinformatics, 80(8), 1974–1986. 10.1002/prot.24086 22488760

[46] Pradhan S, Schwartz RA, Patil A, Grabbe S, Goldust M. 2022. Treatment options for leishmaniasis. Clinical and Experimental Dermatology, 47(3), 516–521. 34480806 10.1111/ced.14919

[47] WHO. 2021. Global leishmaniasis surveillance: 2019–2020, a baseline for the 2030 roadmap – Surveillance mondiale de la leishmaniose: 2019–2020, une période de référence pour la feuille de route à l’horizon 2030. Weekly Epidemiological Record = Relevé Épidémiologique Hebdomadaire, 96, 401–419. Available from https://iris.who.int/handle/10665/344795 last access 4 July 2024.

[48] Shadab M, Jha B, Asad M, Deepthi M, Kamran M, Ali N. 2017. Apoptosis-like cell death in *Leishmania donovani* treated with KalsomeTM10, a new liposomal amphotericin B. PLoS One, 12(2), e0171306. 28170432 10.1371/journal.pone.0171306 PMC5295687

[49] Shamas-Din A, Brahmbhatt H, Leber B, Andrews DW. 2011. BH3-only proteins: Orchestrators of apoptosis. Biochimica et Biophysica Acta (BBA) Molecular Cell Research, 1813(4), 508–520. 21146563 10.1016/j.bbamcr.2010.11.024

[50] Smirlis D, Duszenko M, Ruiz AJ, Scoulica E, Bastien P, Fasel N, Soteriadou K. 2010. Targeting essential pathways in trypanosomatids gives insights into protozoan mechanisms of cell death. Parasites & Vectors, 3, 107. 21083891 10.1186/1756-3305-3-107 PMC3136144

[51] Smirlis D, Soteriadou K. 2011. Trypanosomatid apoptosis: “Apoptosis” without the canonical regulators. Virulence, 2, 253–256. 21566464 10.4161/viru.2.3.16278

[52] Studer G, Rempfer C, Waterhouse AM, Gumienny R, Haas J, Schwede T. 2020. QMEANDisCo – distance constraints applied on model quality estimation, Bioinformatics, 36(6), 1765–1771. 31697312 10.1093/bioinformatics/btz828 PMC7075525

[53] Uliana SR, Trinconi CT, Coelho AC. 2018. Chemotherapy of leishmaniasis: present challenges. Parasitology, 145(4), 464–480. 28103966 10.1017/S0031182016002523

[54] Yagoubat A, Corrales RM, Bastien P, Lévêque MF, Sterkers Y. 2020. Gene editing in trypanosomatids: tips and tricks in the CRISPR-Cas9 era. Trends in Parasitology, 36(9), 745–760. 32703742 10.1016/j.pt.2020.06.005

[55] Yan Y, Tao H, He J, Huang SY. 2020. The HDOCK server for integrated protein–protein docking. Nature Protocols, 15(5), 1829–1852. 32269383 10.1038/s41596-020-0312-x

