# Supplementary material for: Apoptotic proteins in Leishmania donovani: in silico screening, modeling, and validation by knock-out and gene expression analysis
Source: Parasite. 2025 Feb 12;32:9. doi: 10.1051/parasite/2024081 (PMC11825125; doi:10.1051/parasite/2024081)
Supplement: Supplementary file 1 — Supplementary Table 1: Primers included in the study. [file parasite-32-9-s1.pdf]

Supplementary materials

Supplementary Table 1: Primers included in the study

**KO Primers**	Details	Primer name	Primer Sequence
**LdBPK_302600.1 (AAPO)**	Donar F'	AK82	CGTGTAGCTGTTCACCGGTGCGGCCGCAAGgtataatgcagacctgctgc
	Donar R'	AK83	ACCGCACTCCAGCAGTGAGGCGGGTCCGTGccaatttgagagacctgtgc
	5' SEED F'	AK84	gaaattaatacgactcactataggCAACAGAGGAGACGTCTATAgttttagagctagaaatagc
	3' SEED F'	AK85	gaaattaatacgactcactataggCCGCTTTGCAGACTATAAACgttttagagctagaaatagc
	Seq F'	AK86	CAGAGGACCAGTTCAACGTCG
	Seq R'	AK87	GCGATCTGCTGCTGCATTGG
	5' UTR F'	AK90	TCTCTCTACAGCAGTCGCAGC
	3' UTR R'	AK91	GTTCTCTTCTCACCCTTTCGG
**LdBPK_291360.1 (PAPO - I)**	Donar F'	AM35	ACCCACCTTTGTTTTTATTCTGCATCCTTCgtataatgcagacctgctgc
	Donar R'	AM36	CGCTACTCAGCTCGCCTTCGCTTTGCCTCTccaatttgagagacctgtgc
	5' SEED	AM37	gaaattaatacgactcactataggGGGGAATACGGGAGAGTTCAgttttagagctagaaatagc
	3' SEED	AM38	gaaattaatacgactcactataggTCCGCTTCCGCCAATGGACAgttttagagctagaaatagc
	Seq F'	AM39	GCCAGTGCAGGTGAATGAGC
	Seq R'	AM40	ACACAACGGTGGCGATGTCC
	5' UTR F'	AM41	GGGCAAACACGCGAAACGG
	3' UTR R'	AM42	AGGCAGAGGGAGCAGAGAGC
**LdBPK_020680.1 (PAPO - II)**	Donar F'	AM43	ACCGGCCGCTTCACAGCTCTCTCCCGTCTTgtataatgcagacctgctgc
	Donar R'	AM44	GCGAAGGGGCGGTGGTGACGGCGGCAGCGTccaatttgagagacctgtgc
	5' SEED	AM45	gaaattaatacgactcactataggTACGTCGCATCTCGCTCTCGgttttagagctagaaatagc
	3' SEED	AM46	gaaattaatacgactcactataggCCCGCACATGGGAATGCGAAgttttagagctagaaatagc
	Seq F'	AM47	GCTGCCTCATCAAGGACGAGG
	Seq R'	AM48	ACTCGATAGCCGCATCCTGG
	5' UTR F'	AM49	GGCAGTGCACTTCATTTCGGC
	3' UTR R'	AM50	CCACCCATACACCGACAGACG
**LdBPK_221270.1 (AQP)**	Donar F'	AL34	GCTCGCAGACCTCTCTCCTGCAGCGGCGCTgtataatgcagacctgctgc
	Donar R'	AL42	GGAGAAGCGCGAAAGAAATATCAGGAAAACccaatttgagagacctgtgc
	5' SEED	AL36	gaaattaatacgactcactataggTGATGGTGTCCTTTGAACGCgttttagagctagaaatagc
	3' SEED	AL43	gaaattaatacgactcactataggACCTAAACCCACAAATGGAGgttttagagctagaaatagc
	Seq F'	AL44	CCGCAAATCCGAAGAGTGCG
	Seq R'	AL45	ACACCACCGCTAATCGGTCC
	5' UTR F'	AL46	GTGCAGCTCTCACACCATCG
	3' UTR R'	AL47	CAAGTCTCCCGCGATCCTCG
**sgRNA Reverse**	R'	ML42	AAAAGCACCGACTCGGTGCCACTTTTTCAAGTTGATAACGGACTAGCCTTATTTTAACTTgctatttctagctctaaaac
**Donar DNA**			
**Gentamycin**	F'	AG06	GCTCGACGTTGTCACTGAAGC
	R'	AG07	AATATCACGGGTAGCCAACGC
	F'	AF86	CAGAGGACCAGTTCAACGTCG
	R'	AF87	GCGATCTGCTGCTGCATTGG
**Puromycin**	F'	AF88	CCCAGATCGACACATTGAGCG
	R'	AF89	TCATGCACCATGTCCTCGGC
**Knock-In**			
**LdBPK_020680.1 (PAPO - II)**	Donar F'	AM67	GCGTCTCGTTCAGGCCTCCACGGCGCTTCGGTATAATGCAGACCTGCTGC
	Donar R'	AM68	TGCGGATGTCCTCACGGCTGAGGCGGTTAACCAATTTGAGAGACCTGTGC
	5' SEED	AM69	GAAATTAATACGACTCACTATAGGACATGATGATGTCGTCGAGAGTTTTAGAGCTAGAAATAGC
	3' SEED	AM70	GAAATTAATACGACTCACTATAGGGTGTGGCAGCACCGCTGCCCGTTTTAGAGCTAGAAATAGC
**qPCR Primers**			
**LdBPK_302600.1 (AAPO)**	F'	AM79	GTCAGGGCAACCCGAAA
	R'	AM80	TTGAAGACACCACCGCTA
**LdBPK_291360.1 (PAPO - I)**	F'	AM81	GAGTGTACGAGCATCCTG
	R'	AM82	CACGCTTCATCAATAAGGTCA
**LdBPK_020680.1 (PAPO - II)**	F'	AM83	CTGAAGATCGAGGCGGA
	R'	AM84	TGCTTGATCGTCTGGAACA
**LdBPK_221270.1 (AQP)**	F'	AM85	CGCCGAAAGTGTGTTTAC
	R'	AM86	AAGACACCACCGCTAAT
**LdBPK_362480.1 (cGAPDH)**	F'	AM55	ATTCCTCACGCACGAGA
	R'	AM56	GTACACGACGCATTCGATA
**LdBPK_180360.1 (GPI8)**	F'	AM57	GATACATGCCATGCGATT
	R'	AM58	GCAACGAGTACATCTCATTC
